# Phase Transitions of Polarised PVDF Films in a Standard Curing Process for Composites

**DOI:** 10.3390/polym13223900

**Published:** 2021-11-11

**Authors:** Nils Vasic, Julian Steinmetz, Marion Görke, Michael Sinapius, Christian Hühne, Georg Garnweitner

**Affiliations:** 1 Institute of Mechanics and Adaptronics, Technische Universität Braunschweig, 38106 Braunschweig, Germany; m.sinapius@tu-braunschweig.de (M.S.); christian.huehne@tu-braunschweig.de (C.H.); 2 Institute for Particle Technology, Technische Universität Braunschweig, 38104 Braunschweig, Germany; m.goerke@tu-braunschweig.de (M.G.); g.garnweitner@tu-braunschweig.de (G.G.)

**Keywords:** pvdf, electroactive polymer, composite structures

## Abstract

The article reports on the influence of annealing PVDF in an autoclave process on the PVDF phase composition. DSC, FTIR and XRD measurements serve to observe the phase changes in an already stretched, polarised and β-phase rich film. Annealing was conducted between 90 and 185 ${}^{\circ }C$ to cover a broad range of curing processes in an autoclave. The β-phase is found to be stable up to near the melting range at 170 ${}^{\circ }C$. At 175 ${}^{\circ }C$, the non-piezoelectric α-phase dominates and the piezoelectric γ- and γ′-phases appear. The γ-phase grows at elevated temperatures and replaces the β-phase. This observation stresses the importance of developing new methods to reactivate the polarisation after annealing, in particular for the integration of PVDF as a sensor in laminated structures, such as CFRP.

## 1. Introduction

Many objects generate an acoustic sound before total structural failure occurs, such as wooden beams in a mine. However, some human-made structures such as adhesive joints do not give a warning before failure. For these objects, structural health monitoring (SHM) has been developed. Sensors are integrated into structures to detect defects in time before total failure occurs. Piezoelectric materials are able to convert mechanical stress into a measurable electric charge and are therefore applied in SHM [1].

Poly(vinylidene fluoride) (PVDF) is such a piezoelectric material. It is widely used as an electroactive polymer and is especially known for its variety of crystalline phases. Among the five known crystalline phases, the α-, β- and γ-phase are particularly noteworthy [2]. The α-phase is non-piezoelectric, whereas the β- and γ-phases are both piezoelectric. The β-phase is used in sensors due to its stronger polarity. During melt casting of (PVDF) sensors, the α-phase crystallises from the melt. For a phase change to the β-phase, the PVDF must be stretched up to 500%, heated to 90 ${}^{\circ }C$ and polarised with an electric field of 50 kV mm^−1^ [2,3]. The piezoelectric effect of the β-phase depends on the temperature to which it is exposed. Ageing starts at 80 ${}^{\circ }C$ [4], and at 110 ${}^{\circ }C$, the piezoelectric effect is lost. This is the so-called curie temperature [1].

This effect proves relevant when PVDF sensors are to be integrated into carbon fibre reinforced plastic (CFRP) structures. Curing those structures occurs at temperatures up to 185 ${}^{\circ }C$, while the α- and β-phase already melt in a range from 162 to 172 ${}^{\circ }C$ [5]. The sensor hence is melted, and the α-phase crystallises from the melt resulting in the loss of the piezoelectricity. However, an already-cured CFRP structure cannot be stretched as described above to produce the β-phase.

The current research investigates the implementation of PVDF into an epoxy bondline. In a first step, PVDF is co-cured to a CFRP structure at 180 ${}^{\circ }C$. The melting of the PVDF is necessary to achieve a strong adhesion. Next, in the epoxy bondline of two CFRP structures, a PVDF sensor is integrated. The epoxy adhesive is normally cured at 130 ${}^{\circ }C$ [6]. However, it is not yet clear if the sensor builds a strong interface to the co-cured PVDF layers at 130 ${}^{\circ }C$ or if it has to be molten again in order to obtain a thermoplastic weld. The question is thus how the elevated temperatures up to the melting range effect the functionality of the PVDF sensor.

The effect of annealing the PVDF at different temperatures is already reported in detail in the literature. At temperatures below 100 ${}^{\circ }C$, the β-phase shows a higher crystallisation rate than the α-phase. The latter shows a maximum crystallisation rate at 130 ${}^{\circ }C$, which declines until the γ-phase dominates at 160 ${}^{\circ }C$ [7]. A loss of piezoelectricity is observed when raising the annealing temperature to 175 ${}^{\circ }C$. The remnant polarisation of an already stretched and polarised film drops to 8% of the initial polarisation after annealing at 175 ${}^{\circ }C$. In contrast, after annealing at 180 ${}^{\circ }C$, the remnant polarisation is 40% [8]. When a stretched film is annealed at 182 ${}^{\circ }C$ pure crystalline β-phase is observed [9]. As mentioned before, this phase should have been melted at 172 ${}^{\circ }C$ [5]. In both cases, the PVDF-film is rich in β-phase and has been fixed so that a change in length is hindered. The factors of depolarisation have been discussed in earlier studies. The loss of polarisation is linked to the greater mobility of molecular chains. It does not appear that the share of crystalline β-phase on the total crystallinity is important [10]. A key factor seems to be the orientation of dipoles in the molecular chain. Up to 155 ${}^{\circ }C$, the crystallinity remains constant, and the crystalline β-phase does not change to a crystalline α-phase. The phase changes do not seem to be important, while the reorientation of the β-dipoles by 60° seems to be the relevant factor in depolarisation [11]. All these findings have been observed under laboratory conditions and not in practical applications.

In our study, an already stretched, polarised and β-phase-rich PVDF film is annealed in an autoclave. The conducted research will focus on the influence of a standard autoclave process on the phase changes in a commercially available PVDF film. As described above, the piezoelectric phase has been found above the melting temperature [9], and an increase in polarisation has been found when the PVDF is fixed during annealing. This is similar to an autoclave process in that length changes are suppressed due to the hydrostatic pressure. Since the disorientation of the β-dipoles seems to be the main cause of depolarisation [11], there should be a few phase transitions. This leads to the hypotheses of our study: the combination of annealing and pressure in an autoclave stabilises the piezoelectric phase up to the high temperatures needed for curing CFRP structures. If this holds to be true, the implementation of polymeric sensor materials will be more feasible in future manufacturing of CFRP structures.

## 2. Materials and Methods

### 2.1. Materials

A 40 μm PVDF film (Nowofol, Siegsdorf, Germany) is used in the experiments. It is pre-stretched and polarised by an electric field induced for 10 min at a temperature of 90 ${}^{\circ }C$. Due to the interests of the supplier, no further information regarding the material is available. From the foil, PVDF films with dimensions of each 15 × 15 mm are cut out.

### 2.2. Phase Analysis

The prepared PVDF films are analysed via differential scanning calorimetry (DSC), Fourier transformed infrared spectroscopy (FTIR) and X-ray diffraction (XRD) measurements. FTIR and XRD measurements are carried out before and after annealing at the chosen temperature. Phase changes in a film are directly detectable. As the DSC measurements are carried out as a destructive measurement, the PVDF films are only measured after annealing. Ten samples of the original foil are chosen to be measured for the comparison with room temperature (RT).

#### 2.2.1. DSC

For the thermal measurements, a differential calorimeter from Mettler Toledo (Columbus, OH, USA) is used. Samples of the film with a minimum mass of 5 mg are placed in an aluminium crucible and heated in reference to a crucible filled with air over an interval of 0 to 200 ${}^{\circ }C$ and a heating rate of 10 K min^−1^. The total crystallinity $X_{C}$ is determined by measuring the melting enthalpy $H_{m}$, which is put in relation to the melt enthalpy of a total crystalline film $H_{m100\%}$ (104.67 J g^−1^) [12].

(1) $$X_{c}=\frac{H_{m}}{H_{100}}$$

#### 2.2.2. FTIR

Infrared spectra of the samples are measured with a Vertex 70v FT-IR Spectrometer (Bruker, Billerica, MA, USA). An attenuated total reflectance (ATR) technique has been carried out in an interval from 10,000 to 400 cm^−1^ with the use of a diamond crystal. The measured intensity of the infrared rays after interchange with a sample is put in reference to the measured intensity of the infrared rays without interchange. This is expressed as a rate of transition *T* or how much of the infrared rays a sample absorbs. By using the Beer–Lambert Law, the absorbance *A* is calculated.

(2) $$A=-log_{10}\;\cdot \;T$$

For the α-phase, the absorption bands at the wavenumbers of 614 and 763 cm^−1^ are observed. The fraction of β-phase has been determined by the absorption band at 1275 cm^−1^, whereas the γ-phase shows absorption bands of 833 and 1234 cm^−1^. A peak accountable to both electroactive phases can be found at 840 cm^−1^. The fraction of the electroactive phase $F_{EA}$ in a film can be determined by

(3) $$F_{EA}=\frac{A_{EA}}{\left(\frac{K_{840}}{K_{763}}\right)A_{763}+A_{EA}}\;\cdot \;100$$

where *A* is the absorbance at the specified wave-numbers, and $K_{840}$ ($7.7\times 10^{4}$ cm${}^{2}$ mol^−1^) and $K_{763}$ ($6.1\times 10^{4}$ cm${}^{2}$mol^−1^) are the absorbance coefficients [5].

#### 2.2.3. XRD

For determination of the crystal structure, the Empyrean CU LEF HR Goniometer (PANalytical, Kitmondo, England) with a Copper-$K_{\alpha }$ radiation of a wavelength of 1.5406  Å is used. The reflected radiation is detected with the Empyrean Series 2 PIXcel${}^{3D}$ with a precision of 0.01° in terms of 2θ. The films have been measured in the Bragg–Brentano arrangement on a silicon carrier. The measured intensity of reflection over 2θ is analysed via the software MestReNova (Version 10.0.2-15465). The β-phase shows an intense reflection at 20.75° and a second smaller reflection at 36.6°. The α-phase shows prominent reflections at 17.7°, 18.4° and 19.9°. Those can be easily interchanged with the γ-phase reflections at 18.5°, 20.1° and 20.3° [13]. It shall be said that the reported β-peaks are different throughout the literature. The peak is also reported to appear at 20.3° [14,15] as well as at 20.6° [16]. In this study, the peaks reported by [13] are chosen as references, which will be discussed in Section 3.3. The share of β-phase of the total crystallinity is determined by the deconvolution of the measured intensity curve. The area underneath the deconvoluted reflections of the β-phase is compared with the total area underneath all crystalline reflections. The share of the area underneath all β-phase reflections is the share of crystalline β-phase in the total crystallinity [17].

### 2.3. Autoclave Process

**Figure 1:**
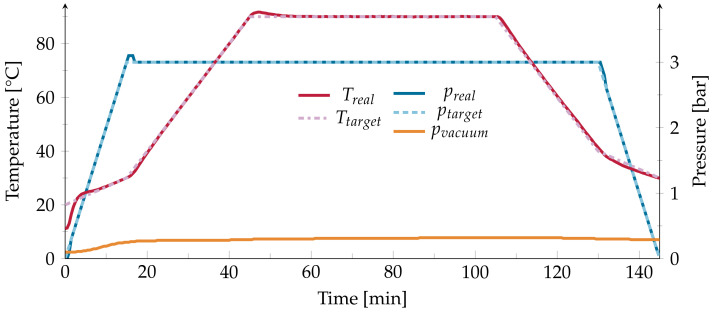
Protocol of the temperature *T* and pressure *p* for an annealing temperature of 90${}^{\circ }C$.

Four PVDF films are placed between two copper plates and sealed in a vacuum bag with approximately 0.3 mbar pressure. As such, the PVDF films will receive an evenly distributed pressure during the experiments. A standard autoclave process for annealing CFRP structures is induced. This process was chosen to stay as close to the later use-case as possible while conducting the research. The temperature is raised from 20 to 30 ${}^{\circ }C$, while the pressure in the autoclave (Scholz SE-510) is raised to 3 bar. The temperature is then raised by 2 K min^−1^ to the desired value. After an hour of annealing, the temperature is lowered by 2 K min^−1^ to 40 ${}^{\circ }C$. Over the course of 15 min, the temperature is lowered to 30 ${}^{\circ }C$ and the pressure is lowered to the ambient pressure (Figure 1). Previous research has been made on the influence of the annealing time and heating as well as cooling rates [5]. As this study focuses on the influence of a standard curing process, no alterations of the mentioned influences will be made.

The values for the annealing temperatures are taken from the autoclave process of a bonded CFRP structure, as well as relevant properties of the PVDF:

90 ${}^{\circ }C$
  The PVDF is usually stretched, polarised and annealed at this temperature to increase the quantity of the β-phase [2,3,18].
130 ${}^{\circ }C$
  The adhesive joint is cured at this temperature in an autoclave process as described above.
160 to 175 ${}^{\circ }C$
  The melting range of α- and β-PVDF is from 167 to 172 ${}^{\circ }C$ [5]. As the melting points of both phases are not clearly separable, a wide space around the melting range is observed.
180 and 185 ${}^{\circ }C$
  CFRP structures are often cured in these temperature ranges.

## 3. Results

### 3.1. DSC

Based on the DSC measurements, a total crystallinity of 55% and a melting point of 171 ${}^{\circ }C$ can be observed in the untreated film at RT. After annealing, the crystallinity decreases in all treated films. The lowest degree of 47% crystallinity is observed after annealing at 90 ${}^{\circ }C$. Overall, the crystallinity fluctuates around 50% (Table 1).

**Figure 2:**
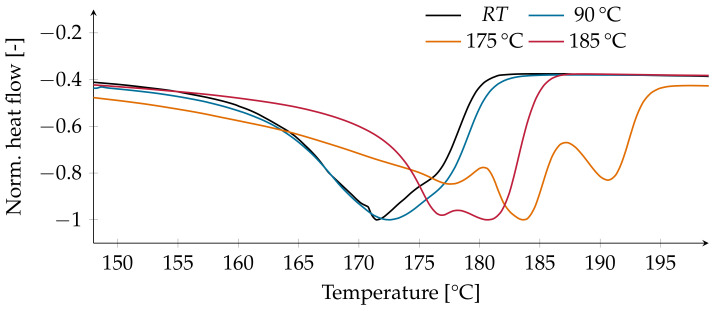
Normalized heat flow over the temperature.

The observed melting point for the α- and β-phase rises from 171 to 178 ${}^{\circ }C$ after annealing at the highest temperatures. For temperatures above 175 ${}^{\circ }C$, a second melting point at 182 ${}^{\circ }C$ appears, which most likely corresponds to the γ-phase [5]. After annealing at 175 and 180 ${}^{\circ }C$, a third melting peak appears at 191 ${}^{\circ }C$ (Figure 2). This may be attributed to the γ′-phase, which crystallizes directly from the α-phase close to the melting point [5].

### 3.2. FTIR

Due to the infared measurements, the total crystalline and amorphous content of the electroactive phase $F_{EA}$ in the untreated film at RT can be calculated to 64%. Only the α- and β-phase are detectable up to an annealing temperature of 170 ${}^{\circ }C$. In this case, $F_{EA}$ equals the total share of the β-phase $F_{\beta }$. The films annealed at 160 ${}^{\circ }C$ show a small increase, whereas $F_{\beta }$ decreases slightly in all films. Above 175 ${}^{\circ }C$, the γ-phase appears and a decrease in $F_{EA}$ to only 35% is calculated. At higher temperatures, $F_{EA}$ decreases only by 4 to 8%. It is not possible to distinguish the β- from the γ-phase (Table 2).

**Figure 3:**
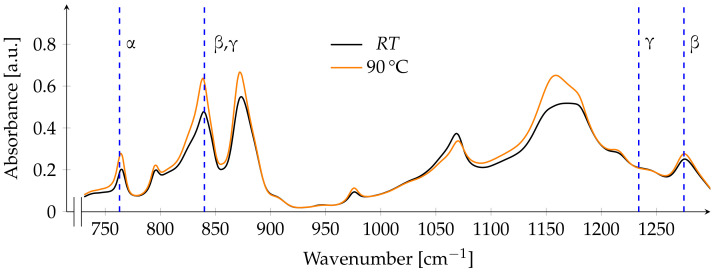
FTIR spectrum before and after annealing at 90 ${}^{\circ }C$. Dashed blue lines indicate the main absorption bands of the respective phases at $A_{763}$, $A_{840}$, $A_{1234}$ and $A_{1275}$.

After annealing at lower temperatures, there is only a small overall increase in absorbance. The peaks indicating the β-phase at 840 and 1275 cm^−1^ show a higher increase than the peak of the α-phase at 763 cm^−1^. The γ-phase is not present in the film, as there is no absorption band at 1234 cm^−1^ (Figure 3).

**Figure 4:**
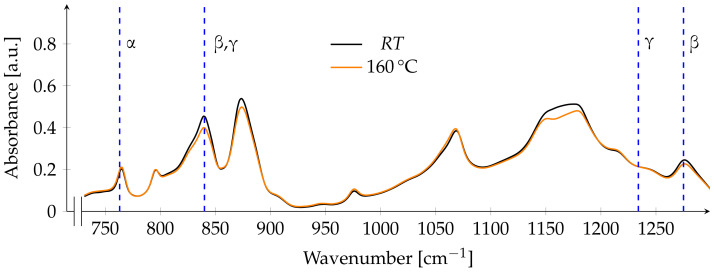
FTIR spectrum before and after annealing at 130 ${}^{\circ }C$. Dashed blue lines indicate the main absorption bands of the respective phases at $A_{763}$, $A_{840}$, $A_{1234}$ and $A_{1275}$.

After annealing at 130 ${}^{\circ }C$, the fraction of $F_{\beta }$ was calculated to be the lowest of all films with 62%. Nearly all peaks show a decrease in absorbance, the only exception to this is the peak referring to the α-phase at 763 cm^−1^ (Figure 4).

**Figure 5:**
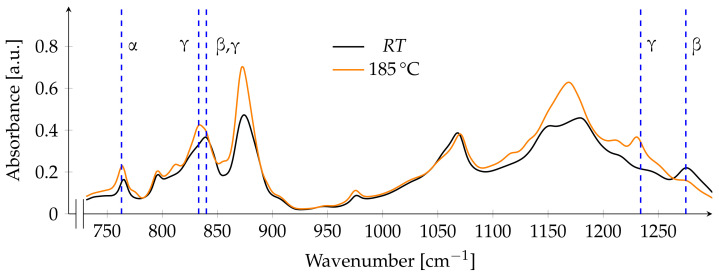
FTIR spectrum before and after annealing at 185 ${}^{\circ }C$. Dashed blue lines indicate the main absorption bands of the respective phases at $A_{763}$, $A_{833}$, $A_{840}$, $A_{1234}$ and $A_{1275}$.

After annealing at the highest temperature, the absorption band assignable to both electroactive phases shifts towards a peak at 833 cm^−1^, which is unique to the γ-phase. An absorption band at 1234 cm^−1^ is apparent, which is also unique to the γ-phase, while the peak related to the β-phase decreases. The peak related to the α-phase also increases slightly (Figure 5). A distinction between the proportion of the β-phase and γ-phase is possible by calculating the peak-to-valley height ratio between the peak related to the γ-phase at 1234 and nearest valley at 1225 cm^−1^, as well as a comparison between the peak related to the β-phase at 1275 and the nearest valley at 1260 cm^−1^. Those height differences multiplied with the total fraction of the electroactive phase $F_{EA}$ are used for calculating the relative fraction of each phase [16]. Due to the poor manifestation of the β-phase absorption band, such comparison is not practicable in this case. However, the observations hint to a dominance of the γ-phase over the β-phase (Figure 5).

### 3.3. XRD

From the XRD patterns, the share of the crystalline β-phase of the total crystallinity in the untreated film is calculated to 88% at RT. At an annealing temperature of 90 ${}^{\circ }C$, the share increases slightly by 2%, while at elevated temperatures, the share decreases (Table 3). The determination is not possible at temperatures above 175 ${}^{\circ }C$, as the observed peaks cannot be assigned to the specific phases.

**Figure 6:**
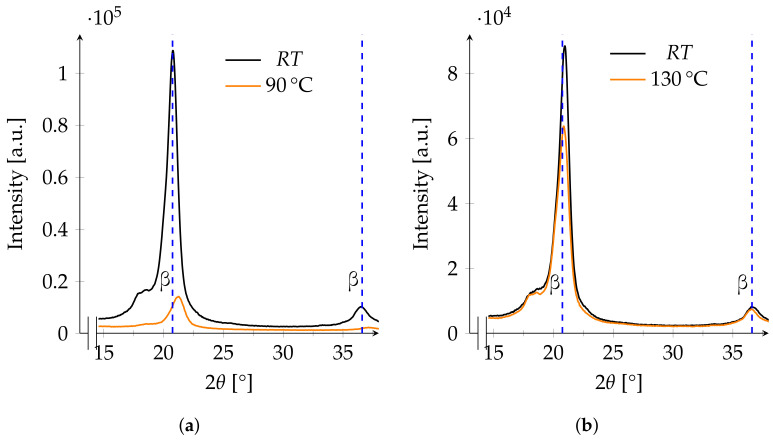
XRD patterns of two films annealed at 90 ${}^{\circ }C$ (**a**) and at 130 ${}^{\circ }C$ (**b**) before and after annealing. The blue dashed lines at 20.75° and 36.6° refer to the expected main reflections of the β-phase.

The two main reflections of the β-phase in the untreated films were detected between 20.8° and 21.3°, as well as between 36.5° and 37°. This will be taken as the reference for the assignment of a peak to the β-phase in this study. An interchange with the γ-phase is unlikely, as there is no trace of the γ-phase detectable in the DSC measurements of the untreated films (Figure 2) nor in the FTIR measurements (Figure 3). The peaks reported by [13] are chosen as references. This is due to the appearance of both β-peaks at 20.75° and 36.6° while measuring at RT. In contrast, the peak of 20.3° for the β-phase reported by [14] does not seems to fit with the conducted measurements at RT. The XRD pattern of one of the films annealed at 90 ${}^{\circ }C$ shows a plateau at 18.1° prior to annealing, which refers to the α-phase. The prominent reflection for the β-Phase at 20.8° moves to 21.2° after annealing, and the intensity is reduced (Figure 6a). The second smaller reflection of the β-phase shifts from 36.5° to 37.2° after annealing. In contrast, after annealing at 130 ${}^{\circ }C$, the peaks of the β-phase stay at 20.9° and 36.6°, while the intensity is only slightly reduced (Figure 6b).

**Figure 7:**
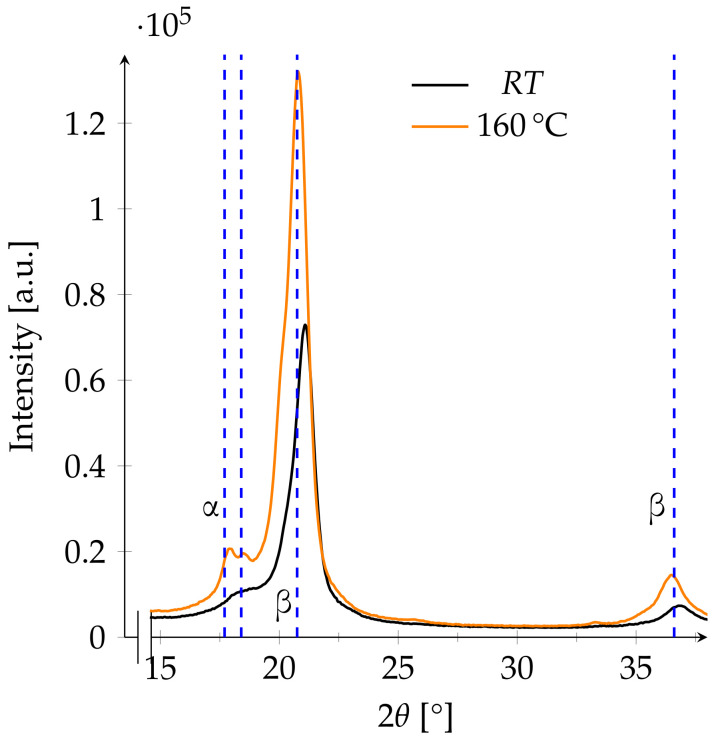
XRD pattern of a film annealed at 130 ${}^{\circ }C$ before and after annealing. The blue dashed lines at 17.7° and 18.4° refer to the expected main reflections of the α-phase, and the lines at 20.75° and 36.6° refer to those of the β-phase.

The sample annealed at 160 ${}^{\circ }C$ shows an increase in intensity after annealing. The reflections of the β-phase are more visible, and the peak of the β-phase shifts from 21.1° to 20.8° (Figure 7).

**Figure 8:**
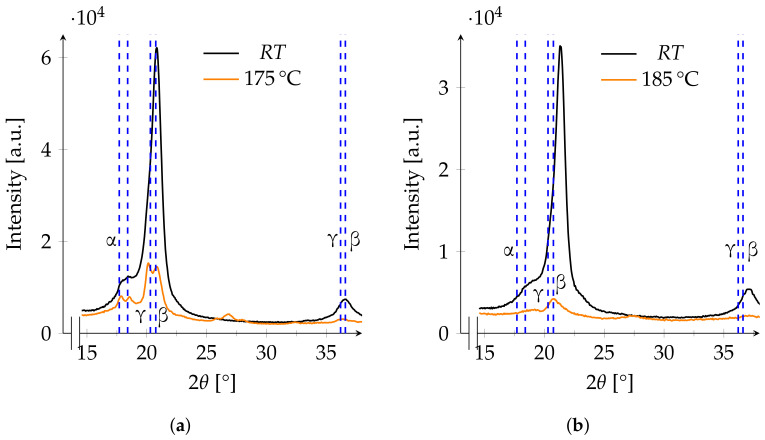
XRD patterns of two films annealed at 175 ${}^{\circ }C$ (**a**) and 185 ${}^{\circ }C$ (**b**) before and after annealing. The blue dashed lines at 17.7° and 18.4° refer to the expected main reflections of the α-phase, while the lines at 20.75° and 36.6° refer to those of the β-phase and the lines at 20.3° and 36.2° to those of the γ-phase.

At temperatures above 175 ${}^{\circ }C$, the assignment of reflections to a specific phase becomes problematic. After annealing, two broad peaks are visible between 20° and 21°, which may be assigned to the β- or γ-phase. Due to the earlier observed shifting of the β-peaks, it cannot be excluded that the reflection at 20.8° is the shifted γ-peak at 20.3°. The reflection observed to be a β-peak at 36.6° in the untreated film shifts to 36.4° after annealing. The γ-phase shows a reflection at 36.2°, a shift by 0.4° fits for both peaks to be assigned to the γ-phase. At the current state, there is no way to distinguish the β- from the γ-phase (Figure 8a). At the highest annealing temperature of 185 ${}^{\circ }C$, the intensity is further reduced so that no clear assignment of peaks to a specific phase can be conducted (Figure 8b).

### 3.4. Phase Changes

The combination of the three described methods enables an estimation of the amorphous and crystalline quantity of the α- and β-phases up to an temperature of 170 ${}^{\circ }C$. The share of the β-phase on the crystallinity can be calculated via the XRD and DSC. Before treatment, 50% of the film is accounted for the crystalline β-phase. The share decreases after treatment, especially at 90, 165 and 170 ${}^{\circ }C$. At 170 ${}^{\circ }C$, the overall crystallinity decreases, and the amorphous fraction increases (Table 4).

For annealing at a temperature above 170 ${}^{\circ }C$, no calculation of the quantity of a specific phase is possible. This is due to the appearance of the γ-phase.

## 4. Discussion

Annealing at temperatures lower than the melting range leads to small interchanges between the β- and α-phase. The total crystallinity varies around 50% and does not show a large decrease. This coincides with already reported fractions of crystallinity [2,19]. The content of the crystalline β-phase decreases only slightly, and most of the crystalline portion transforms to the amorphous phase. There does not seem to be an excessive exchange between β- and α-phase. According to previous studies, the α-phase shows its maximum crystallisation rate at 130 ${}^{\circ }C$ [7]. The here observed total quantity of α-phase changes from 35% to 38% after annealing at 130 ${}^{\circ }C$. Even though the α-phase grows the strongest below the melting point, such a small exchange between the phases alone cannot explain the loss of polarisation after the Curie temperature is reached. The decrease in polarisation may be linked to a loss of orientation of the dipoles in the β-phase, which rotate by 60° after annealing above the Curie temperature [11]. In the Bragg–Brentano arrangement used for the XRD measurements, only those lattice planes parallel to the surface can be detected. If the planes are now disoriented and rotated by 60°, the intensity in the XRD patterns should decrease. This effect can be observed in the XRD patterns, for example, when annealing at 130 ${}^{\circ }C$, as shown in this study. It supports the theory [11] that the loss of β-phase is only a part of the depolarisation and the loss of orientation is at least equally important. Moreover, it implies that stabilising the orientation of the dipoles in an already pre-stretched, polarised and β-phase rich PVDF sensor may help to keep the piezoelectric effect while annealing close to the melting range. Finding a method to stabilise the β-phase with the help of an electric field is an ongoing study. The reason behind the increase in intensity at 160 ${}^{\circ }C$ is presently unknown. In all four films annealed at 160 ${}^{\circ }C$, the intensity increases, whereas in all other films, the intensity decreases after annealing.

At higher temperatures, the γ-phase replaces the β-phase. As observed earlier, when annealing a pre-polarised sample with a high ratio of β-phase up to 175 ${}^{\circ }C$, the polarisation is reduced to 2% and recovers to 8% of the original value after cooling. In contrast, at an annealing temperature of 180 ${}^{\circ }C$, the polarisation recovers from 1% to 40% of the original value [8]. This has not yet been linked to the crystallisation of the γ-phase so far. Concerning the observed phase changes in this study, it is likely that the recovery of polarisation is due to the crystallisation of the γ-phase. Moreover, the observation of pure crystalline β-phase can not be reproduced [9]. This is due to the fact that the γ- and β-phases both show close reflections in the XRD patterns that are used to assign the phases. Nevertheless, piezoelectric phases are present after annealing at high temperatures. The γ-phase shows a higher thermal stability as the melting point is around 182 ${}^{\circ }C$ and the melting point of the γ′-phase is at around 192 ${}^{\circ }C$ (Table 1). Further research should cover if utilising the γ-phase can enhance the integration of PVDF in the SHM of epoxy bondlines.

## 5. Conclusions

In this paper, we have shown that when annealing PVDF in an autoclave process at temperatures lower than the melting range, the β-phase is stable up to 170 ${}^{\circ }C$. Phase changes occur above the melting point as the β-phase is diminished by the crystallisation of the γ-phase. The hypothesis has been shown to be true—the combination of annealing and pressure in an autoclave process stabilises the piezoelectric phases up to temperatures as high as 185 ${}^{\circ }C$. Both piezoelectric phases are likely to be disoriented and therefore require polarisation. This observation stresses the importance of developing new methods of reactivating the polarisation after annealing and the importance of research on how to activate and utilise the γ-phase. In view of these findings, it seems possible to embed PVDF sensors while manufacturing CFRP structures in an autoclave process and keep the functionality of the sensor. Whether these sensors use the β- or γ-phase and how the orientation is stabilised or reactivated with the aid of an electric field are challenges of future research.

## Figures and Tables

**Table 1:** Calculated crystallinity *X* and observed melting points [n${}_{RT}$ = 10, n${}_{90\;\mathrm{to}\;185\;{}^{\circ }C}$ = 4].

Temperature (${}^{\circ }$C)	$X_{\mathrm{total}}$ (%)	Melting Point (%)
RT	55.2 ± 2.6	171.2 ± 1.0
90	46.6 ± 2.9	171.2 ± 1.5
130	52.5 ± 7.3	171.4 ± 1.0
160	54.9 ± 6.3	172.2 ± 1.4
165	48.4 ± 3.3	174.4 ± 1.6
170	48.9 ± 3.5	175.8 ± 0.3
175	49.2 ± 3.3	177.2 ± 0.7, 183.2 ± 1.3, 190.8 ± 0.3
180	48.3 ± 4.1	177.5 ± 0.2, 181.8 ± 0.4, 193.0 ± 0.4
182	49.4 ± 3.5	177.7 ± 0.3, 181.4 ± 0.7
185	50.9 ± 3.8	177.1 ± 0.2, 180.7 ± 0.2

**Table 2:** Calculated fraction of the electroactive phases $F_{EA}$ (Equation (3)) and observed phases (n = 4).

Temperature (${}^{\circ }$C)	$F_{{\mathrm{EA}}_{\mathrm{RT}}}$ (%)	$F_{{\mathrm{EA}}_{\mathrm{annealed}}}$ (%)	Phases${}_{\mathrm{RT}}$	Phases${}_{\mathrm{annealed}}$
90	63.8 ± 2.0	62.5 ± 1.6	α, β	α, β
130	64.6 ± 1.4	61.6 ± 1.9	α, β	α, β
160	63.0 ± 2.2	65.1 ± 1.8	α, β	α, β
165	65.2 ± 5.3	63.0 ± 2.2	α, β	α, β
170	64.2 ± 4.2	62.8 ± 3.1	α, β	α, β
175	64.5 ± 1.4	35.2 ± 3.0	α, β	α, β, γ
180	64.8 ± 1.9	57.0 ± 3.4	α, β	α, β, γ
182	61.7 ± 1.8	51.0 ± 9.3	α, β	α, β, γ
185	63.5 ± 2.7	59.3 ± 2.4	α, β	α, β, γ

**Table 3:** Share of the crystalline β-Phase of the total crystallinity in the film (n = 4).

Temperature (${}^{\circ }$C)	$X_{\beta_{\mathrm{RT}}}$ (%)	$X_{\beta_{\mathrm{annealed}}}$ (%)
90	87.4 ± 1.4	90.6 ± 3.2
130	88.2 ± 1.8	87.7 ± 4.4
160	87.8 ± 2.1	86.6 ± 1.8
165	87.4 ± 2.9	87.0 ± 1.0
170	89.0 ± 1.8	85.5 ± 0.8

**Table 4:** Share of crystalline *X* and amorphous *Y* α- and β-phase (n${}_{RT}$ = 10, n${}_{90\;\mathrm{to}\;170\;{}^{\circ }C}$ = 4).

Temperature (${}^{\circ }$C)	$X_{\beta }$ (%)	$Y_{\beta }$ (%)	$X_{\alpha }$ (%)	$Y_{\alpha }$ (%)
RT	49.6 ± 0.1	14.7 ± 2.2	5.6 ± 0.3	30.1 ± 2.2
90 ${}^{\circ }C$	42.3 ± 2.0	20.3 ± 3.6	4.4 ± 1.7	33.1 ± 2.6
130 ${}^{\circ }C$	46.2 ± 8.0	15.5 ± 7.4	6.3 ± 2.1	32.0 ± 3.3
160 ${}^{\circ }C$	47.6 ± 5.7	17.5 ± 4.4	7.4 ± 1.2	27.6 ± 1.9
165 ${}^{\circ }C$	42.1 ± 2.8	21.0 ± 3.4	6.3 ± 0.8	30.7 ± 2.8
170 ${}^{\circ }C$	41.8 ± 3.1	20.9 ± 5.1	7.1 ± 0.6	30.1 ± 2.9

## Data Availability

The raw data of the experiments can be requested from the authors.

## Abbreviations

The following abbreviations are used in this manuscript:

ATR	attenuated total reflectance
CFRP	carbon fibre reinforced plastic
DSC	differential scanning calorimetry
FTIR	Fourier transformed infrared spectroscopy
PVDF	poly(vinylidene fluoride)
RT	room temperature
SHM	structural health monitoring
XRD	X-ray diffraction
